# Psoriatic microRNAs induce NK cell activation via an innate immune crosstalk abrogated by the Toll-like receptor 7/8 antagonist Enpatoran

**DOI:** 10.1186/s12967-026-07909-5

**Published:** 2026-03-02

**Authors:** Carolina Gaudenzi, Irene Lo Cigno, Helena Stabile, Roberta Maggio, Francesca Bianchi, Gaia Giongrandi, Stefania Zini, Tiziana Schioppa, Laura Tiberio, Angela Ceribelli, Carlo Selmi, Marisa Gariglio, Angela Gismondi, Silvano Sozzani, Annalisa Del Prete, Flavia Bazzoni, Daniela Bosisio, Valentina Salvi

**Affiliations:** 1 Department of Molecular and Translational Medicine, Università degli Studi di Brescia, Viale Europa 11, 25123 Brescia, Italy; 2 Department of Translational Medicine, University of Eastern Piedmont, Novara, Italy; 3 Department of Molecular Medicine, Sapienza University of Rome, Rome, Italy; 4 Officina Farmaceutica, Good Manufacturing Practice Facility, IRCCS Ospedale Pediatrico Bambino Gesù, Rome, Italy; 5 Department of Biomedical Science for Health, University of Milan, Milan, Italy; 6 Laboratorio Morfologia Umana Applicata, IRCCS Policlinico San Donato, Milan, Italy; 7 Department of Rheumatology and Clinical Immunology, IRCCS Humanitas Research Hospital, Milan, Italy; 8 Department of Biomedical Sciences, Humanitas University, Pieve Emanuele, Milan, Italy; 9 Department of Molecular Medicine, Sapienza University of Rome, Laboratory Affiliated to Institute Pasteur-Italia, Rome, Italy; 10 Department of Medicine, University of Verona, Verona, Italy

**Keywords:** NK cells, microRNAs, pDCs, Monocytes, TLRs, Psoriasis

## Abstract

**Background:**

MicroRNAs (miRNAs) are short regulatory RNAs that can be released in extracellular vesicles and, under pathological conditions such as autoimmunity, activate innate immune cells through Toll-like receptor (TLR) 7 and 8. This mechanism may sustain chronic inflammation. Psoriasis is an immune-mediated skin disease where the role and activation pathways of Natural Killer (NK) cells remain incompletely understood. We investigated whether miRNAs upregulated in psoriatic lesions contribute to NK cell activation.

**Methods:**

A pool of psoriasis-associated miRNAs (pso-miR) was generated and used to stimulate either purified NK cells or peripheral blood mononuclear cells (PBMCs). NK cell activation was assessed in terms of cytokine secretion and target cell killing. Inhibitor experiments were performed to demonstrate TLR activation by pso-miR.

**Results:**

Pso-miR did not directly activate NK cells, which lack TLR7/8, but triggered NK effector functions, including IFN-γ secretion and cytotoxicity, within PBMCs, indicating the involvement of accessory cells. Mechanistically, pso-miR engaged TLR7/8-expressing plasmacytoid dendritic cells and monocytes, leading to the secretion of IFN-α, IL-12, and IL-18. These cytokines, in turn, drove full NK cell activation. We also identified a previously overlooked subset of CD56^dim^ NK cells in psoriatic skin, representing mature cytotoxic NKs. Moreover, pso-miR stimulation of PBMCs induced IFN-γ-producing CD8^+^ T cells, further amplifying tissue-damaging responses.

**Conclusions:**

Altogether, these findings reveal that psoriatic miRNAs activate an innate immune loop, which indirectly drives NK cell and CD8^+^ T cell effector functions via TLR7/8-dependent cytokine signalling, representing a novel pathogenic mechanism of psoriatic inflammation and keratinocyte damage. Of note, such miRNA-mediated crosstalk is abrogated by the dual TLR7/8 antagonist Enpatoran, highlighting a therapeutic avenue for modulating immune activation in psoriasis.

**Supplementary Information:**

The online version contains supplementary material available at 10.1186/s12967-026-07909-5.

## Introduction

MicroRNAs (miRNAs) are short, noncoding, single strand RNAs (ssRNAs) originally discovered as post-transcriptional regulators of gene expression within producing cells [1, 2]. Soon after, miRNAs were found to transfer to neighboring cells, mostly via extracellular vesicles (EVs), suggesting potential cell-to-cell regulatory functions [3–5]. Beyond canonical RNA interference, extracellular miRNAs can act noncanonically by triggering ssRNA-sensing innate immune receptors, namely Toll like receptor 7 and 8 (TLR7/8) [6–8].

TLRs are pattern recognition receptors typically expressed by innate cells which alert the body about infections by recognizing conserved molecular components of pathogens and igniting inflammation [9]. TLRs also recognize endogenous ligands released by stressed or damaged tissues (i.e. under sterile conditions) to promote immune-mediated tissue repair. However, TLR activation needs tight spatio-temporal regulation, since prolonged or unnecessary inflammation paves the way to chronic inflammatory and autoimmune diseases [10]. Among human TLRs, TLR7 and TLR8 are both localized in the endosomes and are activated by short ssRNA sequences derived from endosomal digestion of longer ssRNAs enriched in guanosine (G) and uridines (U) [6, 11–16]. However, their expression and functional significance within innate immune responses are distinct [11]: while TLR7 is expressed by plasmacytoid dendritic cells (pDCs) and masters the production of IFN-α, TLR8 is expressed by conventional DCs, monocytes and neutrophils and induces inflammatory cytokines [17]. The activation of endosomal TLRs by endogenous ligands and consequent unwanted inflammation plays a central role in the induction and progression of several immune-mediated inflammatory diseases, such as psoriasis, systemic lupus erythematosus and rheumatoid arthritis [18–21]. In this regard, our group and others showed that selected extracellular miRNAs upregulated in psoriasis, rheumatoid arthritis but also cancer, sepsis and neurodegeneration, could induce the production of pathogenetic interferon (IFN)-α by pDCs via human TLR7 [22, 23] or inflammation via TLR8 [24–26]. Of note, the blocking of TLR activation by self-nucleic acids via specific inhibitors, such as the dual TLR7/8 antagonist Enpatoran, represents a promising therapeutic strategy in the near future [20, 27, 28].

Psoriasis is a systemic immuno-mediated inflammatory disease characterized by distinctive skin lesions [29]. The initiation of the autoreactive immune response depends on the aberrant production of type I IFNs by TLR7-espressing pDCs [30–32], which accumulate in psoriatic skin [21, 33, 34], where they are triggered by self-nucleic acids released by damaged keratinocytes [35–37]. Several miRNAs are dysregulated in psoriasis, both at the cellular and extracellular levels [38–41], and can activate pDCs [23]. Following this initiation phase, autoimmune tissue damage is sustained by an auto-amplifying cytokine loop involving IFN-γ, TNF-α and IL-17 [42–45]. While this loop highlights the role of adaptive cells, namely T helper 17 and T helper 1 lymphocytes, recent evidence shows that innate immune cells beyond pDCs also contribute to disease pathogenesis [46].

Natural killer (NK) cells are innate lymphoid cells capable of discriminating infected and tumor cells from normal counterparts using receptors belonging to the killer cell immunoglobulin-like receptor and C-type lectin-like receptor families [47–49]. Target cells are eliminated via granzyme release and Fas ligand expression [50–52]. Besides cytotoxicity, NK cells also produce cytokines, with IFN-γ being the most distinctive [50]. NK cells also express some TLRs, such as TLR3, while the expression and function of TLR7/8 remains debated [53–58]. Two major NK cell subtypes can be distinguished based on CD56 marker expression: CD56^dim^ NK, accounting for over 90% of all peripheral blood NK cells and expressing cytotoxic proteins including CD16 (often referred to as circulating NK cells), and CD56^bright^ NK cells, primarily present in peripheral tissues but scarce in the blood and specialized in IFN-γ production (often referred to as tissue-resident NK cells) [59]. Although NK cells are increased in psoriatic skin lesions [60–62], their mechanisms of activation and contribution to disease remain poorly defined [63, 64].

This work was designed to determine whether extracellular miRNAs upregulated in psoriatic skin may activate NK cells, thereby representing a novel pathogenetic mechanism of immune activation and tissue damage.

## Material and methods

### Cell preparation and culture

Peripheral blood mononuclear cells (PBMCs) were obtained by density gradient centrifugation and maintained in RPMI 1640 medium supplemented with 10% heat-inactivated, endotoxin-free FBS, 2 mM L-glutamine, penicillin and streptomycin (complete medium; all from Gibco, Thermo Fisher Scientific). To obtained highly purified NK cells and total monocytes, CD3-depleted PBMCs (Miltenyi Biotec) were labeled with anti-CD56-APC (clone HCD56 Biolegend), anti-CD3-VioGreen (clone REA613, Miltenyi Biotec), anti-CD16-FITC (clone REA423, Miltenyi Biotec), anti-CD14-PerCP-Vio700 (clone REA599, Miltenyi Biotec), anti-HLA-DR-VioBlue (clone REA805, Miltenyi Biotec) and sorted on a FACSAria III cell sorter (BD). In the CD3^-^ HLA-DR^-^ population, CD56^bright^CD16^-^ and CD56^dim^CD16^+^ cells were sorted as NK cells, while in the CD3^-^CD56^-^HLA-DR^+^ population, CD14^+^CD16^-^, CD14^+^CD16^+^ and CD14^±^CD16^+^ cells were sorted as total monocytes. The purity of sorted population was routinely 99.0–99.9%. pDCs were obtained from PBMCs after negative immunomagnetic separation with the Plasmacytoid Dendritic Cell Isolation kit II (Miltenyi Biotec). Purified cells were cultured in completed RPMI medium.

### Cell stimulation

Cells were stimulated with 10 $\mu$g/ml of psoriatic miRNA mix (pso-miR) composed of synthetic hsa-miR203a-3p (87.3%), miR142-3p (8.9%), miR146a-5p (3%), miR574-5p (0.6%), miR32-5p (0.05%), miR187-3p (0.05%), miR3911 (0.05%) and miR1180-3p (0.05%). All synthetic miRNAs were stabilized with a phosphorothioate linkage between each base (Integrated DNA Technologies). Complexation of pso-miR with DOTAP (Roche Diagnostics) was performed as previously described [23, 26]. Whenever indicated, cells were pretreated for 1 hour with 1 $\mu$M Enpatoran (Enp, MedChemExpress), 1 $\mu$g/ml of B18R (Thermo Fisher Scientific), 1 $\mu$g/ml of an anti-human-IL12 p70 (clone 24,910, R&D Systems), 1 $\mu$g/ml of an anti-human-IL15 (clone 34,583, R&D Systems), 1 $\mu$g/ml of an anti-human-IL18 (clone 125-2 H, R&D Systems) or 1 $\mu$g/ml of an anti-human-BDCA2 antibody (clone AC144, Miltenyi Biotec).

### Analysis of CD69 expression and intracellular IFN-$\gamma$ production by flow cytometry

PBMCs or sorted NK cells were stimulated with pso-miR for 24 hours. Brefeldin A (5 µg/mL, Merck) was added to cultures during the last 5 hours of stimulation. Cells were stained for surface markers using anti-CD3-VioBlue (clone REA613, Miltenyi Biotec), anti-CD4-VioGreen (clone REA623, Miltenyi Biotec), anti-CD16-FITC (clone REA423, Miltenyi Biotec), anti-CD69-PE-Vio770 (clone FN50, Miltenyi Biotec), anti-CD56-APC (clone HCD56, Biolegend) and anti-CD8-APC-Vio770 (clone REA734, Miltenyi Biotec). Cells were then fixed and permeabilized using the Inside Stain kit (Miltenyi Biotec) and stained with an anti-IFN-γ-PE antibody (clone 45–15, Miltenyi Biotec). Samples were read on a MACSQuant 16 analyzer (Miltenyi Biotec) and analyzed using the FlowJo Software (BD).

### Analysis of NK cell cytotoxicity

A fixed number of K562 target cells (15,000), previously labelled with 250 nM cell Trace Violet (Thermo Fisher Scientific), were incubated with increasing numbers of pso-miR-activated effector cells (PBMCs or sorted NK cells). After incubation for 4 hours, Propidium Iodide (PI, Invitrogen) was added, and dead target cells were identified as Cell Trace Violet^+^ PI^+^ by flow cytometry.

### CD107a degranulation assay

PBMCs, sorted NK cells alone or co-cultured with autologous pDCs and total monocytes were stimulated with pso-miR for 24 hours and then incubated with K562 target cells at the ratio 1:1. After 1 hour, 50 $\mu$M monesin (Merck) was added to this coculture for an additional incubation of 3 hours.

The cells were stained with the following antibodies: anti-CD3-VioGreen (clone REA613, Miltenyi Biotec), anti-HLA-DR-VioBlue (clone REA805, Miltenyi Biotec), anti-CD16-FITC (clone REA423, Miltenyi Biotec), anti-CD56-APC (clone HCD56, Biolegend), and anti-CD107a-PE (clone H4A3, Miltenyi Biotec). Samples were read on a MACSQuant 16 Analyzer (Miltenyi Biotec) and analyzed with FlowJo (BD).

### qPCR of mRNAs and miRNAs

RNA was extracted using TRIzol reagent and treated with DNAse according to the manufacturer’s

instructions, and reverse transcription was performed using random hexamers and Moloney Murine Leukemia Virus Reverse Transcriptase (MMLV RT) (all from Thermo Fisher Scientific). The SsoAdvanced Universal SYBR Green Supermix (Bio-Rad) was used according to the manufacturer’s instructions. The following primers were used: IFN-$\alpha$ fw 5’-CCTCCTGTCTGATGGACAGAC-3’, IFN-$\alpha$ rev 5’-GGTTGAAGATCTGCTGGATCAGC-3’, IFN-$\alpha$ fw 5’-CAGCAATTTTCAGTGTCAGAAGC-3’, IFN-$\beta$ rev 5’-TCATCCTGTCCTTGAGGCAGT-3’, IL-12 p35 fw 5’-GAATGTTCCCATGCCTTCACCAC-3’, IL-12 p35 rev 5’-CAATGGTAAACAGGCCTCCACTG-3’, IL-15 fw 5’-CAGATAGCCAGCCCATACAAG-3’, IL-15 rev 5’-GGCTATGGCAAGGGGTTT-3’, IL-18 fw 5’-GTCCAGCATTGGAAGTGACC-3’, IL-18 rev 5’-AGGCCACACAGGATAAGCTC-3’, IFN-$\gamma$ fw 5’-GCAGGTCATTCAGATGTAGCGG-3’, IFN-$\gamma$ rev 5’-CCACACTCTTTTGGATGCTCTGG-3’, TLR7 fw 5’-TTAACCAATTGCTTCCGTGTC-3’, TLR7 rev 5’-GGTGCCCACACTCAATCTG-3’, TLR8 fw 5’-TGTGGTTGTTTTCTGGATTCAA-3’, TLR8 rev 5’-GCTCGCATGGCTTACATGA-3’, HPRT fw 5’-CCAGTCAACAGGGGACATAAA-3’and HPRT rev 5’-CACAATCAAGACATTCTTTCCAGT-3’. Gene expression was normalized based on HPRT mRNA content.

For miRNA amplification from psoriatic skin, the miRCURY LNA RT Kit and the miRCURY LNA miRNA Probe PCR assay in association with miRCURY SYBR Green PCR Kit (all from Qiagen) were used. To monitor the miRNA extraction efficacy and for miRNA normalization, the RNA spike-in cel-miR39 was added to Qiazol reagent before miRNA extraction.

Reactions were run in triplicate on a StepOne Plus Real-Time PCR System (Applied Biosystems) and analyzed by StepOne Plus Software (Version 2.3, Applied Biosystems).

### Cytokine analysis

IL-12 p70, IL-15, IL-18, and IFN-γ were measured by ELISA (R&D Systems). IFN-α was detected using specific Module Set ELISA kit (Invitrogen, BMS216MST). IFN-β was measured by a bioluminescence kit (Invivogen, luex-hifnbv2). All assays were performed on cell-free supernatants according to the manufacturer’s protocol.

### Western blot analysis

NK cells, pDCs, monocyte-derived DCs (moDCs) and HEK293-transfected cells were lysed in NP40/Triton X-100 lysis buffer (10 mM Tris-HCl, pH 7.9; 150 mM NaCl; 0.6% NP-40; 0.5% Triton X-100), supplemented with inhibitors (1 mM Na_3_VO_4_, 2 mM DTT, 1 mM NaF, 1 mM PMSF, and protease inhibitor cocktail; all from Millipore Sigma). Equal amounts of extracts were analyzed through SDS-PAGE followed by Western blotting with antibodies against TLR7 (rabbit monoclonal,

5632, Cell Signaling Technology), TLR8 (rabbit monoclonal, 11,886, Cell Signaling Technology), and β-actin (mouse monoclonal, C4, sc-47778, Santa Cruz Biotechnology). Protein bands were detected with SuperSignal West Pico Chemiluminescent Substrate (Pierce).

### Human samples

Matched lesional and non-lesional (i.e. normal-appearing, distant from the plaques) skin and PBMCs were obtained from adult psoriatic patients with early lesions (<10 d), devoid of any previous topical or systemic pharmacological treatment. Samples were taken after informed consent and approval of the Ethics Committee of the Humanitas University (Milan, Italy) and in accordance with the Declaration of Helsinki. Punch biopsy specimens (4 or 6 mm) were collected, mechanically dissociated, and digested with 1.5 μg of type I DNase and 24 μg of type I-A collagenase (both from Sigma-Aldrich, St. Louis, MO, USA) in 5 mL of RPMI medium for 30 minutes at 37 °C. Cell suspensions were subsequently purified by Lymphoprep density gradient centrifugation and immediately processed for flow cytometric analysis. Alternatively, tissues were used for RNA extraction as described above.

### Statistical analysis

Sample group normality was confirmed by Shapiro-Wilk test before application of parametric statistical analysis. Statistical significance among the experimental groups was determined using 2-tailed unpaired Student’s t test or 1-way ANOVA with Dunnett’s post hoc or Tukey test (GraphPad Prism 10) as indicated in each figure legend. P less than 0.05 was considered significant; n indicates the number of biological replicates and is specified in each figure legend.

## Results

### Several GU-rich TLR7/8 activating miRNAs are upregulated in psoriatic skin lesions

**Fig. 1:**
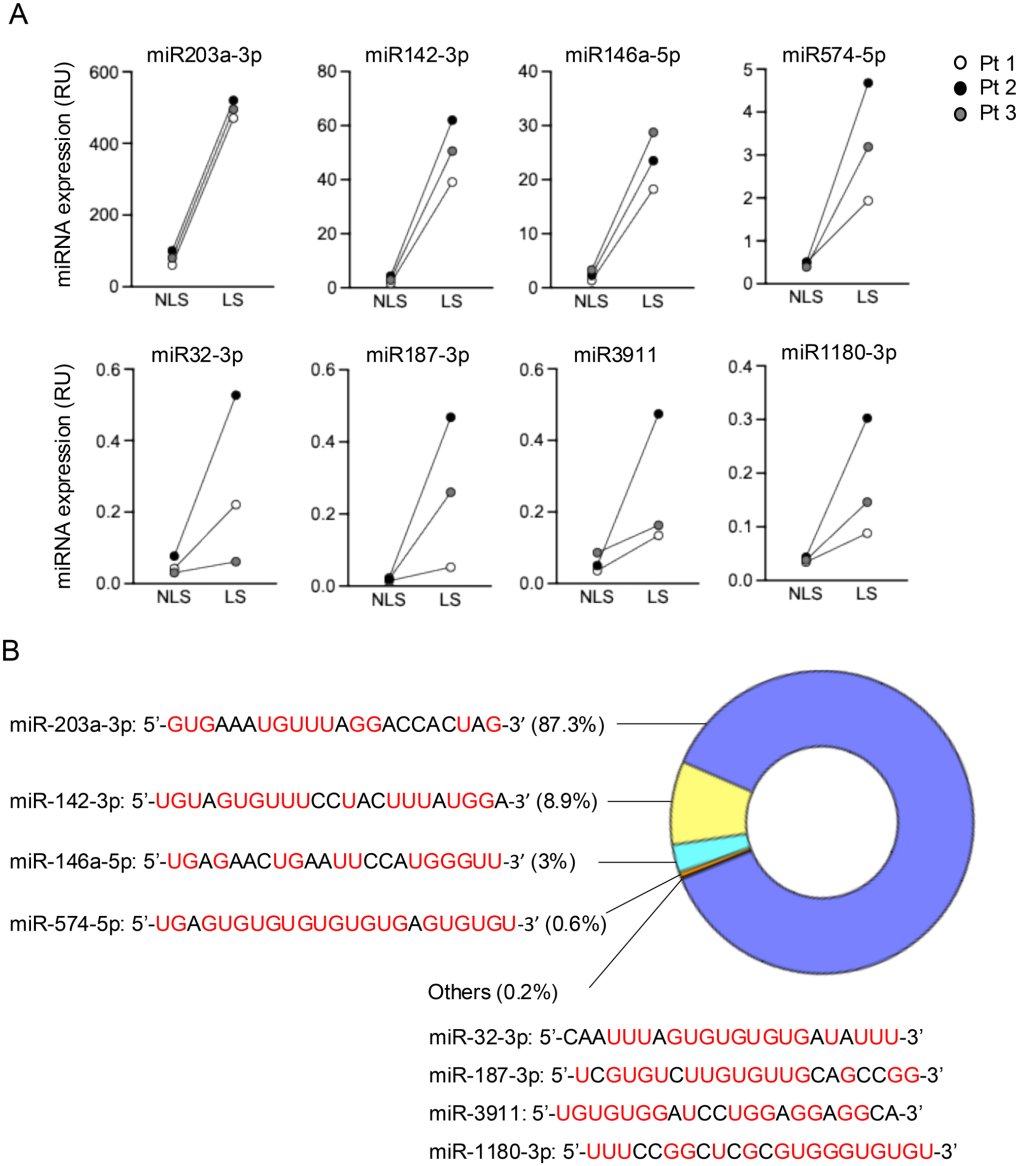
Gu-rich miRNAs upregulated in psoriatic skin lesions were used to prepare a miRNA mix (pso-miR) for cell stimulation. (**A**) Expression of the indicated miRNAs was investigated by real-time PCR in paired non-lesional (NLS) and lesional skin (LS) biopsies from 3 psoriatic patients. Each dot represents an individual patient, and lines connected paired NLS and LS. miRNA expression is reported as relative units (RU) normalized to cel-miR39. (**B**) Pso-miR composition. For each miRNA, the sequence (G and U highlighted in red) and percentage within the mix are indicated

Previous literature, including our own work, demonstrated a consistent increase of miR203a-3p in psoriatic skin lesions and also in the plasma of psoriatic patients compared to healthy donors, showing that it is released upon skin damage and accumulates extracellularly [23, 38, 39, 65]. By investigating the expression of other GU-rich miRNAs in these same biopsies, we detected a marked upregulation in lesional skin of miR142-3p, miR146a-5p, miR574-5p, miR32-3p, miR187-3p, miR3911 and miR1180-3p in addition to miR203a-3p (Fig. 1A). Results are shown in terms of relative expression as compared to cel-miR39, thus allowing to estimate the level of expression of each miRNA. In the figure, miRNAs are arranged from the most highly expressed (miR203a) to the least expressed (miR1180). Of note, several of these miRNAs were also found in extracellular vesicles released by damaged keratinocytes described in a previous work from this group [23], suggesting that miRNAs other than miR203a can act as cell-to-cell communicators.

Based on these results, we prepared a mix of synthetic miRNAs (pso-miR) in which each miRNA contributed in a percentage representative of that observed in lesional psoriatic skin (Fig. 1B).

### Pso-miR triggers NK cell activation within PBMCs via TLR7/8

**Fig. 2:**
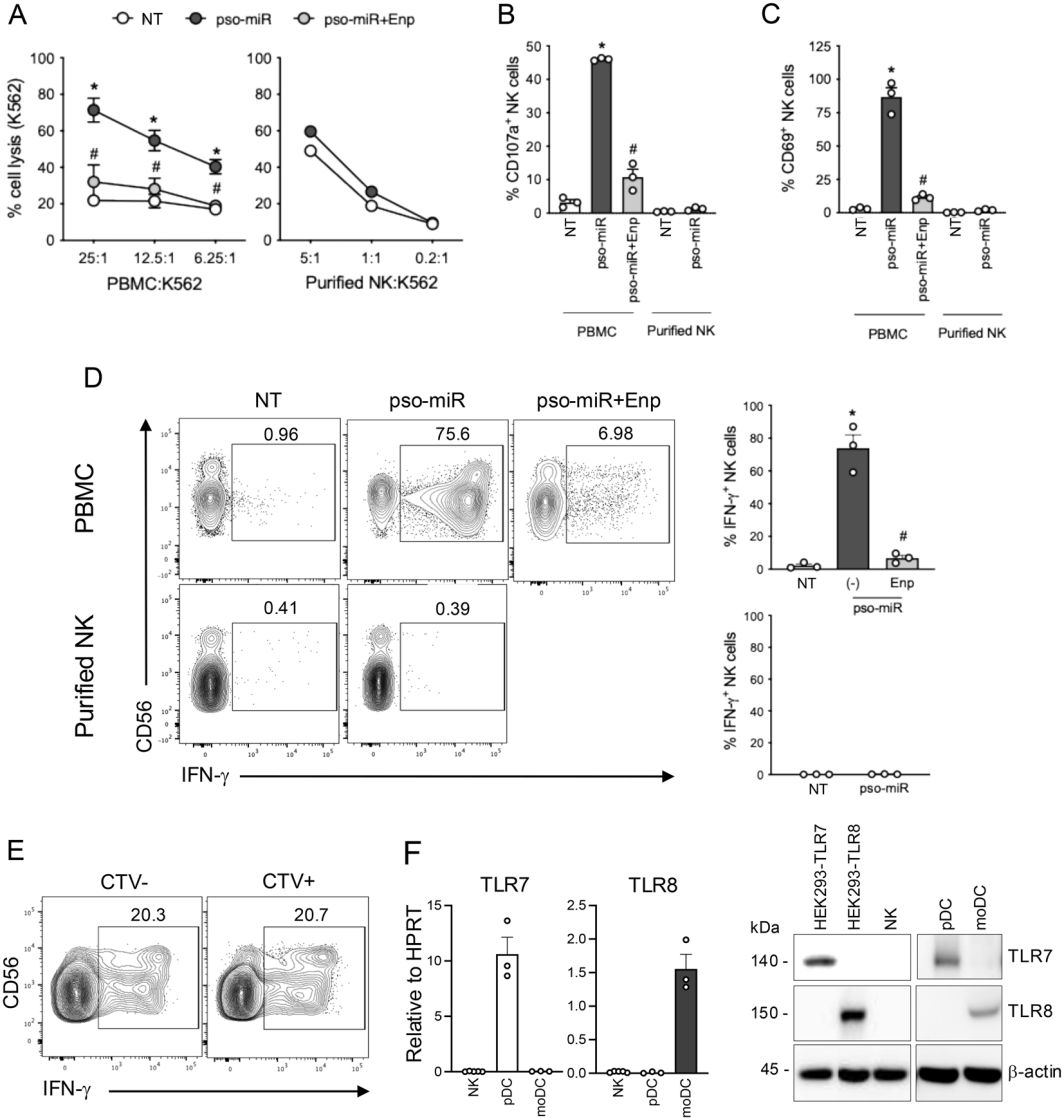
NK cell activation by pso-miR depends on TLR7/8-expressing accessory cells. (**A**-**D**) PBMCs or highly purified NK cells were stimulated with pso-miR (10 $$\mu $$g/ml) or with vehicle alone (NT) for 24 hours. Where indicated, PBMCs were pretreated with Enpatoran (enp, 1 $$\mu $$M) for 1 hour. (**A**) After stimulation, PBMCs or highly purified NK cells were cocultured with NK-sensitive K562 cells for 4 hours at the indicated cell ratio. Data are expressed as mean ± SEM (*n* = 3) of the percentage of K562 cell lysis. (**B**) PBMCs or highly purified NK cells were stimulated as in A and then cocultured with K562 cells at a 1:1 ratio. CD107a expression was assessed by flow cytometry. Data are expressed as mean ± SEM (*n* = 3) of the percentage of CD107a$$^ + $$ NK cells. (**C**) Surface expression of CD69 on CD56^+^CD3^–^ NK cells was assessed by flow cytometry. Data are expressed as mean ± SEM (*n* = 3) of the percentage of CD69 NK cells. (**D**) for the analysis of intracellular IFN-$$\gamma $$, brefeldin a (5 $$\mu $$g/ml) was added for the final 5 hours of culture. Left, representative flow cytometry graphs showing intracellular staining of IFN-$$\gamma $$ in CD56^+^CD3^–^ NK cells in PBMCs (upper panels) or purified NK cells (lower panels). Right, bar graphs from three independent experiments. Data are expressed as mean ± SEM (*n* = 3) of the percentage of IFN-$${\gamma ^ + }$$ producing NK cells in PBMCs (upper panel) or purified NK cells (lower panel). (A-D) *p < 0.05 versus “NT” and ^#^*p* < 0.05 versus “pso-miR” by one-way ANOVA with Tukey’s post-hoc test. (**E**) Highly purified NK cells were labelled with CellTracker Violet (CTV) and added to autologous PBMCs. IFN-$$\gamma $$ expression in CTV^-^ (untouched) and CTV^+^ (previously purified) NK cells was assessed by flow cytometry after 24 hours of stimulation with pso-miR. One representative experiment out of three is shown. (**F**, left panels) real-time PCR showing the expression of TLR7 and TLR8 mRNAs in highly purified NK cells (*n* = 5). pDcs (*n* = 3) and monocyte-derived DCs (moDC, *n* = 3) were used as positive controls of TLR7 and TLR8 expression respectively. Data are expressed as mean ± SEM of 2^-ΔCt^ relative to HPRT. (**F**, right panels) indicated cell types, including HEK293 cells transfected to overexpress TLR7 or TLR8 (HEK293–TLR7 and HEK293–TLR8), were lysed and the expression of TLR7, TLR8 and $$\beta $$-actin was determined by Western blot. One representative fluorogram out of three is shown

To understand whether miRNAs upregulated in psoriatic skin may represent novel pathogenic activators of NK cells, total PBMCs and highly purified primary NK cells were stimulated with pso-miR and assessed for activation in terms of target cell lysis, CD69 expression and IFN-$\gamma$ production in the presence/absence of Enpatoran, a selective TLR7/8 antagonist. Within PBMCs, pso-miR stimulated NK cell cytotoxicity against K562 target cells, a prototypical NK-sensitive leukemia cell line (Fig. 2A, left panel) and NK degranulation as assessed by CD107a expression (Fig. 2B). Both activities were strongly inhibited by Enpatoran, indicating that NK cell activation is mediated by TLR7/8 stimulation. However, pso-miR failed to activate purified NK cells (Fig. 2Aright panel and 2B, right part of the bar graph). Similarly, pso-miR promoted the up-regulation of the activation marker CD69 (Fig. 2C) and IFN-$\gamma$ secretion (Fig. 2Dupper panel) in a TLR7/8 dependent manner, whereas no effect was observed in highly purified NK cells (Fig. 2Cright part of the bar graph and 2D lower panels). To exclude a purification artefact, purified NK cells were labeled with CellTrace Violet (CTV) and added back to autologous PBMCs. As shown in Fig. 2E, CTV^+^NK cells produced IFN-γ in response to pso-miR at levels comparable to “untouched” CTV^-^NK cells, defined as NK cells that were not subjected to any purification or enrichment procedures. In line with our results, we demonstrate that highly purified NK cells do not express TLR7 and TLR8, both at the mRNA (Fig. 2F, left panels) and protein levels (Fig. 2F, right panel), despite previous reports suggesting otherwise [53, 54, 58].

Collectively, our findings demonstrate that NK cell activation by pso-miR occurs indirectly, via crosstalk with accessory cells expressing functional TLR7/8.

### Pso-miR induces TLR7/8-dependent secretion of NK-activating cytokines within PBMCs

**Fig. 3:**
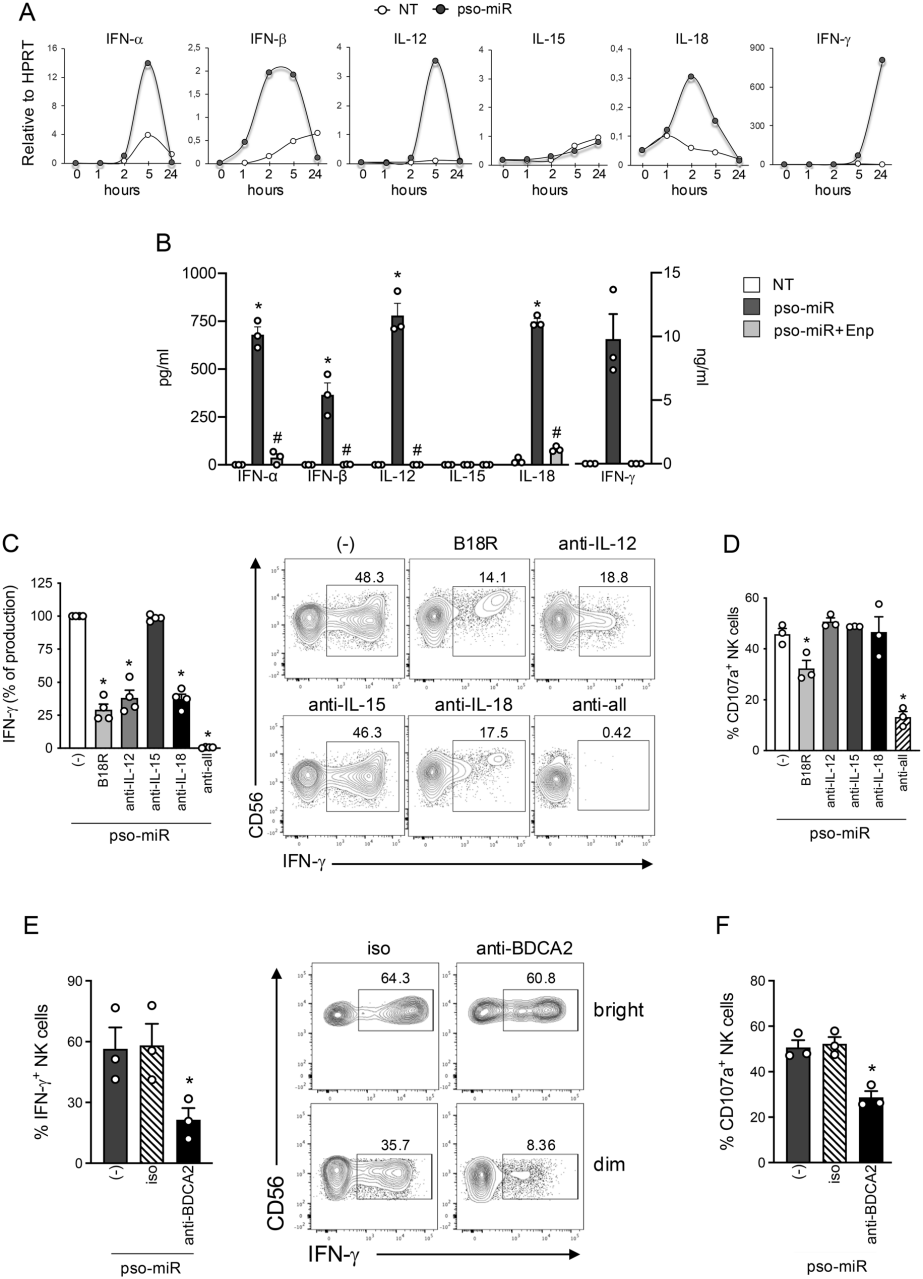
Type I IFNs, IL-12 and IL-18 are the cytokines required for full NK cell activation within PBMCs. (**A**) PBMCs were stimulated for the indicated time-points with pso-miR (10 $$\mu $$g/ml) or with vehicle alone (NT) and mRNA expression levels of the specified cytokines were evaluated by real-time PCR. Data are displayed as 2^-ΔCt^ relative to HPRT from one representative experiment out of three. (**B**) PBMCs were pretreated with Enpatoran (enp, 1 $$\mu $$M) for 1 hour and then stimulated with pso-miR for 24 hours. The production of secreted cytokines was assessed by ELISA. Data are expressed as mean ± SEM (*n* = 3); *p < 0.05 versus “NT” and ^#^*p* < 0.05 versus “pso-miR” by one-way ANOVA with Tukey’s post-hoc test. (C-D) PBMCs were pretreated with specific cytokine blockers (1 $$\mu $$g/ml each) or in combination (anti-all) for 1 hour and then stimulated with pso-miR for 24 hours. (**C**) Left, intracellular IFN-$$\gamma $$ production was evaluated by flow cytometry in CD56^+^CD3^–^ NK cells. Data are expressed as mean ± SEM (*n* = 3) of the percentage of IFN-$$\gamma $$ setting the stimulation with pso-miR (-) as 100%; *p < 0.05 versus “(-)” by one-way ANOVA with Dunnet’s post-hoc test. Right, dot plots from one representative experiment of IFN-$$\gamma $$ staining in CD56^+^CD3^–^ NK cells in PBMCs. (**D**) Surface expression of CD107a on CD56^+^CD3^–^ NK cells was assessed by flow cytometry after 4 hours of coculture with K562 cells at a 1:1 ratio. Data are expressed as mean ± SEM (*n* = 3) of the percentage of CD107a$$^ + $$ NK cells; *p < 0.05 versus “(-)” by one-way ANOVA with Dunnet’s post-hoc test. (E-F) PBMCs were pretreated with anti-BDCA2 antibody or isotype control (both at 1$$\mu $$g/ml) for 1 hour and then stimulated with pso-miR for 24 hours. (E) Left, intracellular IFN-$$\gamma $$ production was evaluated by flow cytometry in CD56^+^CD3^–^ NK cells. Data are expressed as mean ± SEM (*n* = 3) of the percentage of IFN-$${\gamma ^ + }$$ producing NK cells. Right, representative flow cytometry graphs showing intracellular staining of IFN-$$\gamma $$ in CD56^bright^ (upper panels) and CD56^dim^ (lower panels) NK cells. (F) Surface expression of CD107a on CD56^+^CD3^–^ NK cells was assessed by flow cytometry. Data are expressed as mean ± SEM (*n* = 3) of the percentage of CD107a$$^ + $$ NK cells. (E-F) *p < 0.05 versus “(-)” by one-way ANOVA with Dunnet’s post-hoc test

We initially investigated whether pso-miR stimulation of PBMCs induced key NK-activating cytokines, including type I IFNs, IL-12, IL-18 and IL-15 [66]. Fig. 3Ashows that pso-miR rapidly induced the transcription of mRNAs for type I IFNs (IFN-$\alpha$ and IFN-$\beta$), IL-12 and IL-18 but not of IL-15. Interestingly, the mRNA of IFN-γ, which is primarily released by NK cells within PBMCs, was induced with a delayed kinetic, supporting the idea that NK cell activation within PBMCs follows the production of other cytokines. The release of pso-miR-induced cytokines, confirmed at the protein levels, was significantly inhibited in the presence of Enpatoran (Fig. 3B). To demonstrate the involvement of these cytokines in NK cell activation, PBMCs were pre-treated with specific neutralizing molecules alone or in combination and then stimulated with pso-miR. A significant inhibition of IFN-γ production was observed in PBMCs treated with B18R, a decoy receptor for type I IFNs, anti-IL-12, anti-IL-18 and the mix of all blocking reagents, but not with anti-IL-15 (Fig. 3C, left panel). Of note, these inhibitors differentially affected the production of IFN-γ by CD56^dim^ and CD56^bright^ NK cells (Fig. 3C, right panel and Supplemental Fig. 1). Specifically, B18R mainly inhibited IFN-γ production by CD56^dim^ NK cells whereas anti-IL-12 suppressed CD56^bright^ NK cells. Blocking IL-18 had a partial effect on both NK cell subsets, while the combination of these specific blockers dramatically reduced the production of IFN-γ by both subsets. When analyzing NK cell cytotoxic activation in terms of CD107a expression, we found that B18R was the sole inhibitor capable to reduce NK cell degranulation (Fig. 3D), while all inhibitors together cooperated to strongly decrease the expression of CD107a induced by pso-miR (Fig. 3D).

Given the effects of B18R, we exploited a different approach to reduce the levels of type I IFNs in PBMCs, meaning the block of pDCs that are the main producers of these cytokines in response to endosomal TLR stimulation. Such goal can be achieved by engaging an inhibitory receptor exclusively expressed by pDCs [67, 68] via anti-BDCA2 antibodies. The engagement of BDCA2 significantly inhibited the production of IFN-$\gamma$ induced by pso-miR (Fig. 3E, left panel), especially in the CD56^dim^ NK cell subset (Fig. 3E, right panels), in accordance with the results obtained with B18R (Fig. 3C). Similarly, PBMC treatment with anti-BDCA2 antibody reduced the expression of CD107a on NK cell surface (Fig. 3F).

Altogether, these results indicate that type I IFNs, IL-12 and IL-18 are the cytokines secreted by accessory cells in response to TLR7/8 stimulation by pso-miR that are responsible for the indirect activation of NK cells. In addition, experiments with anti-BDCA2 antibodies strongly suggested a role for pDCs as the main type I IFN producers in response to pso-miR.

### pDCs and monocytes respond to pso-miR by secreting key NK-activating cytokines

**Fig. 4:**
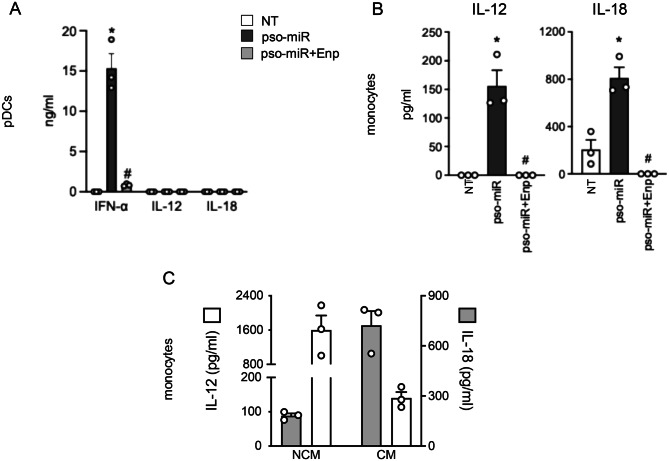
pDCs and monocytes respond to pso-miR by secreting key NK-activating cytokines. (A) Purified pDCs were stimulated with pso-miR (10 $\mu$g/ml) for 24 hours in the presence or absence of Enpatoran (enp, 1 $\mu$M, 1 hour pretreatment), and the secretion of the indicated cytokines was evaluated by ELISA. (B) Total monocytes were sorted and stimulated with pso-miR (10 $\mu$g/ml) in the presence or absence of Enpatoran (enp) for 24 hours. The production of IL-12p70 and IL-18 was evaluated by ELISA in cell-free supernatants. (A-B) Data are expressed as mean ± SEM (*n* = 3); *p < 0.05 versus “NT” and ^#^*p* < 0.05 versus “pso-miR” by one-way ANOVA with Tukey’s post-hoc test. (C) Non-classical (NCM) and classical (CM) monocytes were sorted and stimulated with pso-miR (10 $\mu$g/ml) for 24 hours. Cytokine production was assessed by ELISA. Data are expressed as mean ± SEM (*n* = 3)

Next, we sought to identify accessory cells that are responsible for NK-activating cytokine production. Based on previous results, we stimulated pDCs with pso-miR. According to our previous work with different TLR7-activating miRNAs [22, 23], we confirm that pso-miR induce a strong release of IFN-α by purified pDCs in a TLR7-dependent manner (Fig. 4A). However, pDCs failed to produce IL-12 and IL-18 in response to pso-miR (Fig. 4A), thus indicating the involvement of other accessory cells in the full activation of NK cell effector functions. Because a population of non-classical monocytes was found to colocalize with NK cells in psoriatic skin lesions [69, 70], we investigated the ability of monocytes to respond to pso-miR. Total monocytes produced both IL-12 and IL-18 following pso-miR stimulation in a TLR7/8-dependent manner (Fig. 4B). Of note, we could discern that non-classical monocytes produced the majority of IL-12 whereas classical monocytes were the main producers of IL-18 (Fig. 4C).

Overall, these results indicate that pDCs and monocytes represent the core accessory cells required for NK cell activation by pso-miR.

### pDCs and monocytes are necessary and sufficient accessory cells for full NK cell activation by pso-miR

**Fig. 5:**
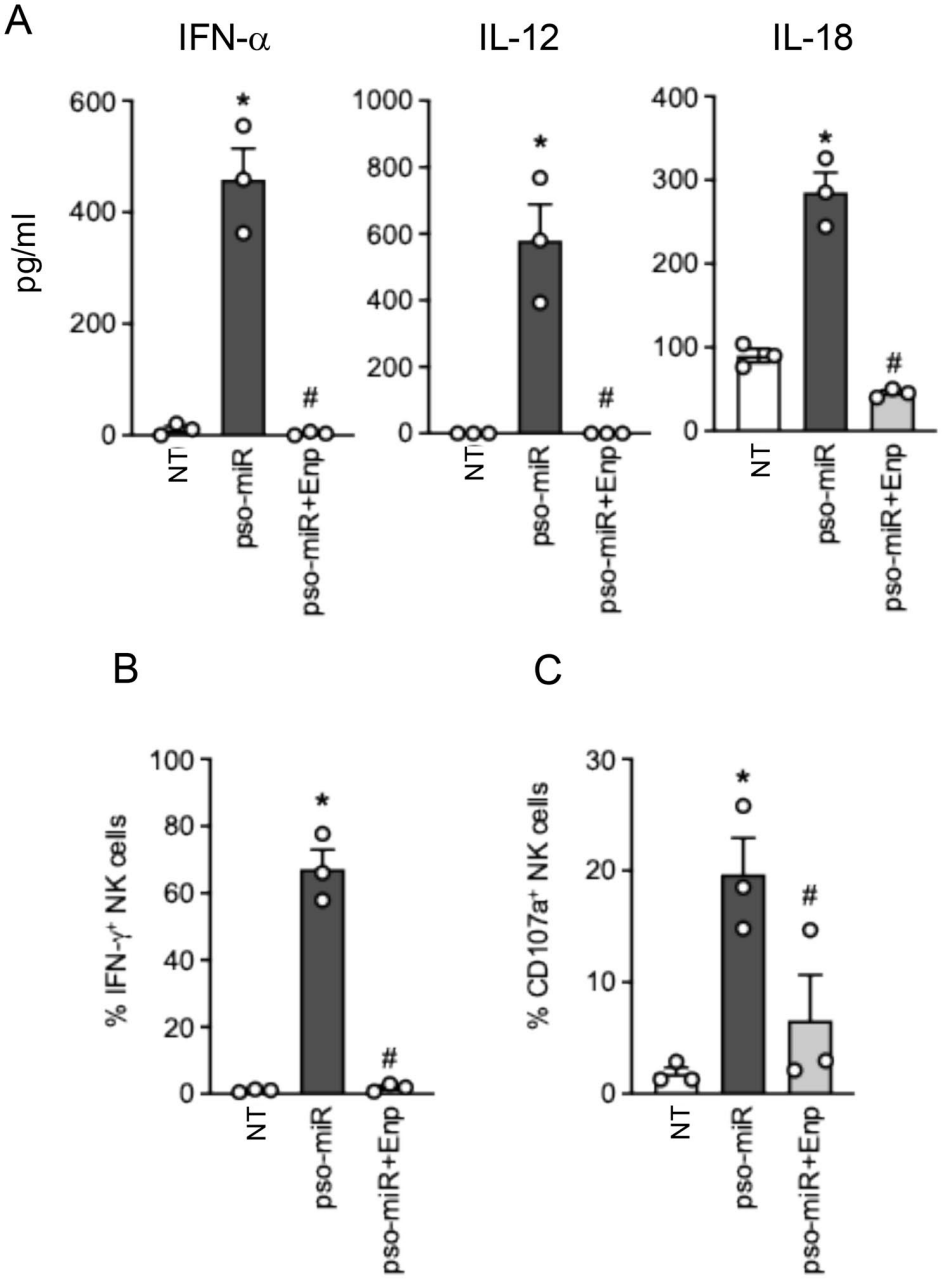
pDCs and monocytes are the accessory cells required for full NK cell activation by pso-miR. (**A**-**C**) Purified pDCs (15,000), sorted NK (150,000) and total monocytes (150,000) were cocultured together and stimulated or not with pso-miR (10 $\mu$g/ml) in the presence or absence of Enpatoran (enp, 1 $\mu$M, 1 hour pretreatment) for 24 hours. (**A**) Cytokine production was evaluated by ELISA in cell-free supernatants. (**B**) Intracellular IFN-$\gamma$ production was evaluated by flow cytometry in CD56^+^CD3^–^ NK cells. (**C**) Surface expression of CD107a on CD56^+^CD3^–^ NK cells was assessed by flow cytometry. (**A**-**C**) Data are expressed as mean ± SEM (*n* = 3). *p < 0.05 versus “NT” and ^#^*p* < 0.05 versus “pso-miR” by one-way ANOVA with Tukey’s post-hoc test

To finally confirm that the full activation of NK cells is the result of the cooperation among pDCs and monocytes, a coculture experiment was performed. Highly purified pDCs, NK cells and total monocytes were cultured together at a 0.1:1:1 ratio, which reproduces the physiological ratio among these cells. First, the production of IFN-α, IL-12 and IL-18 in response to pso-miR was evaluated in cell-free supernatant by ELISA. As shown in Fig. 5A, pso-miR induced the production of all the cytokines in a TLR7/8-dependent manner. In accordance, NK cells cocultured with pDCs and monocytes produced significant amounts of IFN-γ (Fig. 5B) and underwent degranulation (Fig. 5C) upon pso-miR stimulation in a TLR7/8-dependent manner.

Thus, pso-miR efficiently activate NK cell responses via a crosstalk with TLR7/8-expressing innate cells, namely pDCs and monocytes, and involve the TLR7/8-mediated release of type I IFNs, IL-12 and IL-18.

### CD56^bright^ and CD56^dim^ NK cells infiltrate lesional skin of psoriatic patients

**Fig. 6:**
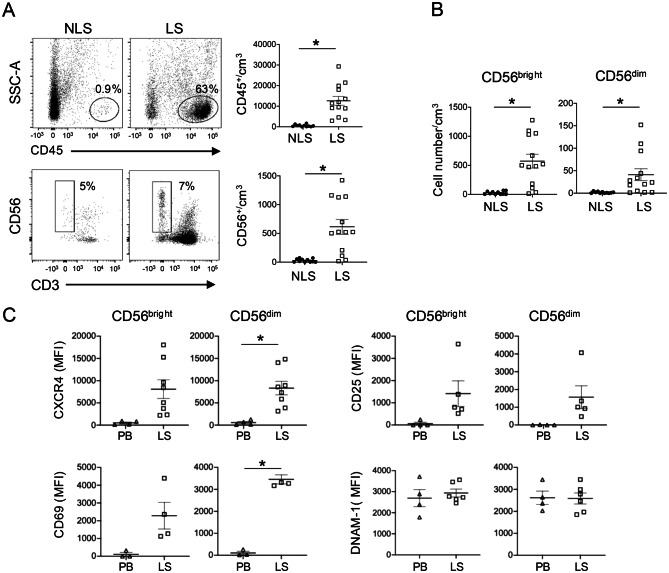
CD56^dim^ NK cells infiltrate psoriatic skin lesions. (**A**) Left. representative gating strategy used to identify the CD56^+^CD3^–^ NK cells within CD45^+^ leukocytes in lesional (LS) and non-lesional skin (NLS) of a psoriatic patient. Right, and (**B**) Median ± SEM of the absolute count of CD45^+^ and CD56^+^CD3 ^–^, CD56^bright^ and CD56^dim^ NK cells in LS (*n* = 13) and NLS (*n* = 11). *p < 0.05 versus “NLS” by unpaired Student’s *t* test. (**C**) Flow cytometric analysis of CXCR4, CD25, CD69 and DNAM-1 expression on CD56^bright^ and CD56^dim^ NK cell subsets isolated from peripheral blood (PB, *n* = 4) or lesions of psoriatic patients (LS, *n* = 5–8). Results are reported as mean ± SEM of the mean fluorescent intensity (MFI) of the indicated markers. *p < 0.05 versus “PB” by unpaired Student’s *t* test

So far, NK cells infiltrating psoriatic lesions were shown to comprise CD56^bright^ cells representing tissue-resident NK cells endowed with regulatory rather than cytotoxic functions, with a possible role in keratinocyte activation via cytokines such as IFN-γ and TNF-α [62]. Based on previous results describing a full activation of NK cells by accessory cells stimulated with pso-miR, we sought to understand whether psoriatic skin lesions may also recruit cytotoxic CD56^dim^ NK cell. In agreement with previous results [62], skin biopsies from lesional psoriatic patients (LS) revealed the presence of huge amount of infiltrating CD45^+^ cells as compared to non-lesional skin (NLS), including CD56^+^CD3^-^ NK cells (Fig. 6A). Among these, we demonstrate the presence of a so far overlooked component of CD56^dim^ NK cells, in addition to the previously described and more abundant CD56^bright^ cells (Fig. 6B). Both subsets expressed at similar levels the skin-homing receptor CXCR4 and the activation markers CD25 and CD69 (Fig. 6C). DNAM-1 is shown as a negative control to demonstrate the specificity of NK cell receptor expression.

Our findings highlight for the first time the presence of a cytotoxic NK population that may contribute to direct tissue damage upon activation by pso-miR, which is not in contrast, but rather complements previous literature, describing psoriasis infiltrating NK to comprise a major component of tissue-resident CD56^bright^ cells endowed with regulatory functions [62].

### The cytokine cocktail induced by pso-miR determines innate activation of a fraction of CD8^+^ T cells in PBMCs

**Fig. 7:**
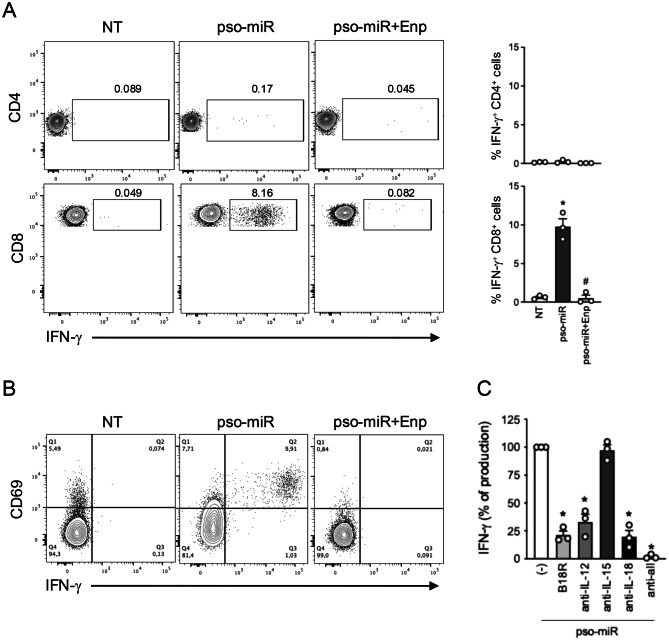
Innate activation of a fraction of CD8^+^ T cells in pso-miR-activated PBMCs. PBMCs were stimulated with pso-miR (10 $\mu$g/ml) or with vehicle alone (NT) for 24 hours, in the presence or absence of Enpatoran (enp, 1 $\mu$M, 1 hour pretreatment). (**A**) For the analysis of intracellular IFN-$\beta$, brefeldin a (5 $\mu$g/ml) was added for the final 5 hours of culture. Left, representative flow cytometry graphs showing intracellular staining of IFN-$\gamma$ in CD3^+^CD4^+^ (upper panels) or CD3^+^CD8^+^ (lower panels) T cells within PBMCs. Right, bar graphs from three independent experiments. Data are expressed as mean ± SEM (*n* = 3) of the percentage of IFN-${\gamma ^ + }$ producing cells. *p < 0.05 versus “NT” and ^#^*p* < 0.05 versus “pso-miR” by one-way ANOVA with Tukey’s post-hoc test. (**B**) Dot plots showing the co-expression of CD69 and IFN-$\gamma$ in CD3^+^CD8^+^ T cells. One representative donor out of three is shown. (**C**) PBMCs were pretreated with specific cytokine blockers (1 $\mu$g/ml each) or in combination (anti-all) for 1 hour and then stimulated with pso-miR for 24 hours. Intracellular IFN-$\gamma$ production was evaluated by flow cytometry in CD3^+^CD8^+^ T cells. Data are expressed as mean ± SEM (*n* = 3) of the percentage of IFN-$\gamma$ production setting the stimulation with pso-miR (-) as 100%; *p < 0.05 versus “(-)” by one-way ANOVA with Dunnet’s post-hoc test

When analyzing the expression of intracellular IFN-γ in PBMCs from healthy donors, we serendipitously noticed that a fraction of CD3^+^CD8^+^ T cells (8%-12%), but not CD3^+^CD4^+^ T cells, accumulated this cytokine (Fig. 7A) and upregulated CD69 (Fig. 7B), both strongly inhibited by Enpatoran. Of note, IFN-γ production was also affected by cytokine inhibitors alone or in combination (Fig. 7C), thus precisely paralleling what was described for NK cell activation. This observation is in line with previous literature describing the capability of virus-specific CD8^+^ T cells to be activated and produce IFN-γ in response to cytokines, especially IL-12, IL-18 and type I IFNs, thus acting as “sentinels” for subsequent, unrelated infections even when their cognate antigen may not be present [71–77]. It is tempting to speculate that these memory CD8^+^ T cells, reactivated by pso-miR-induced cytokines, may further cooperate to the establishment of the psoriatic cytokine milieu.

## Discussion

In this work, we demonstrate that a mixture of GU-rich miRNAs reproducing the miRNA milieu upregulated in psoriatic skin lesions (pso-miR) activates an innate immune loop involving pDCs and monocytes, which secrete cytokines in response to TLR7/8 stimulation. These cytokines, in turn, induce NK cell activation both as IFN-γ-producing cells and as cytotoxic effectors. Importantly, pso-miR does not directly activate NK cells, which do not express TLR7 or TLR8, highlighting the essential role of accessory cells in this process. The presence of activated CD56^dim^ NK cells in psoriatic lesions, representing the mature cytotoxic NK cells subset, supports the in vivo relevance of this pathway and suggests that pso-miR-mediated immune activation may contribute to psoriatic tissue damage through both sustained inflammation and direct keratinocyte cytotoxicity.

The ability of extracellular miRNAs to act as endogenous ligands for endosomal TLR7/8 has emerged as a mechanism of sterile inflammation in autoimmune and chronic inflammatory diseases [7, 8, 22, 25, 26, 56, 78–88]. Work by our own group previously demonstrated that GU-rich miRNAs and miRNA-enriched EVs can activate pDCs and myeloid DCs via TLR7 [22, 23] and TLR8 [26] respectively. We also showed the activity of mixtures of TLR-activating miRNAs, each used at suboptimal concentrations [22, 26], indicating that miRNA pools can activate TLRs regardless of the relatively low concentration of each miRNA. The importance of the pool, rather than of one single deregulated miRNA, was finally confirmed in a more physiological setting where damaged keratinocytes released EVs enriched with hundreds of TLR7-activating miRNAs, which we proposed to work as an EV-associated alarmin cargo contributing to pDC activation in the earliest phases of psoriatic pathogenesis [23]. Despite the experimental limitation of using synthetic miRNAs delivered by DOTAP, the present study reinforces and extends this model by demonstrating that a miRNA pool overexpressed in psoriatic lesions can act as an activator and/or booster of autoimmunity by engaging multiple accessory cell types and indirectly activating NK cells.

The contribution of accessory cells to NK cell activation is well established especially in infections, where NK activation largely depends on the presence of cytokine-producing accessory cells, such as monocytes, macrophages or dendritic cells specialized in pathogen recognition. However, it is obvious that the precise identity of accessory cells involved in each specific response can differ [66]. Using co-culture systems, we demonstrate that pDCs and monocytes represent the key accessory cells responding to pso-miR by secreting IFN-α and IL-12/IL-18, respectively. Although most evidence for accessory cell–dependent NK cell activation derives from infectious models [89–92], our data demonstrate that a comparable mechanism operates in the sterile inflammatory context of psoriasis.

This is particularly relevant from a physiological perspective, as both pDCs and monocytes, including non-classical monocytes, are recruited to psoriatic lesions and are found in close proximity to NK cells, facilitating productive cell-to-cell crosstalk in the inflamed skin microenvironment [34, 69, 93, 94]. Our experiments do not exclude the possible and not contrasting contribution of other TLR7/8 expressing cells, in particular monocyte-derived DCs which express TLR8 and respond to miRNAs by secreting Th1-polarizing cytokines [26], nor the possibility that direct contact with accessory cells, in addition to cytokine production, may contribute to optimal NK cell activation in response to pso-miR [58].

A further layer of complexity is provided by the functional specialization of monocyte subsets. We show that classical and non-classical monocytes display distinct cytokine profiles in response to pso-miR, with classical monocytes preferentially producing IL-18 and non-classical monocytes producing IL-12. These findings are consistent with previous reports describing subset-specific cytokine responses in other inflammatory settings [95]. Importantly, these cytokines differentially regulate NK cell subsets, with IL-12 promoting IFN-γ production by CD56^bright^ NK cells and IL-18, together with IFN-α, preferentially supporting cytotoxic activation of CD56^dim^ NK cells. These results suggest that pDC and classical monocytes may preferentially support NK cytotoxicity, while non-classical monocytes their regulatory role.

The expression of TLR7 and TLR8 in primary NK cells is highly controversial, with both positive [53, 54, 58] and negative [55–57] evidence. These discrepancies may depend on several factors such as different primer sets or antibodies, very small contaminants such as monocytes that confer positivity or responsiveness ([53] and our unpublished results), but also the physiological conditions of NK cells, such as the level of activation in different donors which may induce TLR7/8 expression. Using ultrapure resting NK cells sorted from healthy donors and stringent experimental controls, we demonstrate the absence of TLR7 and TLR8 expression at both the mRNA and protein levels. We also show as Supplemental Fig. 2 that IL-12, IL-18 and IFN-α, at different concentrations and in the presence or absence of R848 (a synthetic TLR7/8 ligand) could not induce the expression of TLR7 or TLR8. In line with these findings, ultrapure NK cells failed to respond directly to pso-miR. These data indicate that circulating NK cells require accessory cell–derived signals to become activated in this context, providing a mechanistic explanation for the indirect nature of NK cell activation observed in our study and reconciling previous reports describing limited or inconsistent NK cell responsiveness to TLR7/8 ligands [53, 54, 58].

In addition to NK cells, we observed cytokine-driven activation of a subpopulation of CD8^+^ T cells, characterized by CD69 upregulation and IFN-γ production, both blocked by Enpatoran. Such “innate-like activation” was previously reported for infections, where, in addition to responding to specific antigens, CD8^+^ T cells can produce IFN-γ in response to cytokines, such as IL-12, IL18 and type I IFNs, elicited by pathogens [71–73]. In the context of psoriasis, this mechanism may further contribute to the establishment and maintenance of a pro-inflammatory cytokine milieu, thereby amplifying immune-mediated tissue damage. Although not the primary focus of this study, these findings suggest that pso-miR–induced cytokine networks may impact multiple lymphocyte populations within psoriatic lesions.

In summary, our study uncovers a novel mechanism of indirect NK cell activation in psoriasis, whereby extracellular miRNAs upregulated in psoriatic skin trigger TLR7/8 on accessory cells. The resulting cytokine milieu drives NK cells, and possibly IFN-γ-producing memory CD8^+^ T cells, into pathogenic effector functions, contributing to autoimmune activation and cytotoxic tissue damage. Of note, this pathogenic circuit is efficiently disrupted by the dual TLR7/8 antagonist Enpatoran, supporting the therapeutic relevance of targeting this pathway in psoriasis. A deeper understanding of these innate immune circuits, together with the ongoing Phase II evaluation of Enpatoran (https://clinicaltrials.gov/study/NCT05162586), will open new perspectives for targeted therapies in psoriasis.

## Electronic supplementary material

Below is the link to the electronic supplementary material.

Supplementary material 1

Supplementary material 2

## Data Availability

All data generated or analysed during this study are included in this published article.
