# Supplementary figures and images for: Psoriatic microRNAs induce NK cell activation via an innate immune crosstalk abrogated by the Toll-like receptor 7/8 antagonist Enpatoran
Source: J Transl Med. 2026 Mar 2;24:478. doi: 10.1186/s12967-026-07909-5 (PMC13059162; doi:10.1186/s12967-026-07909-5)

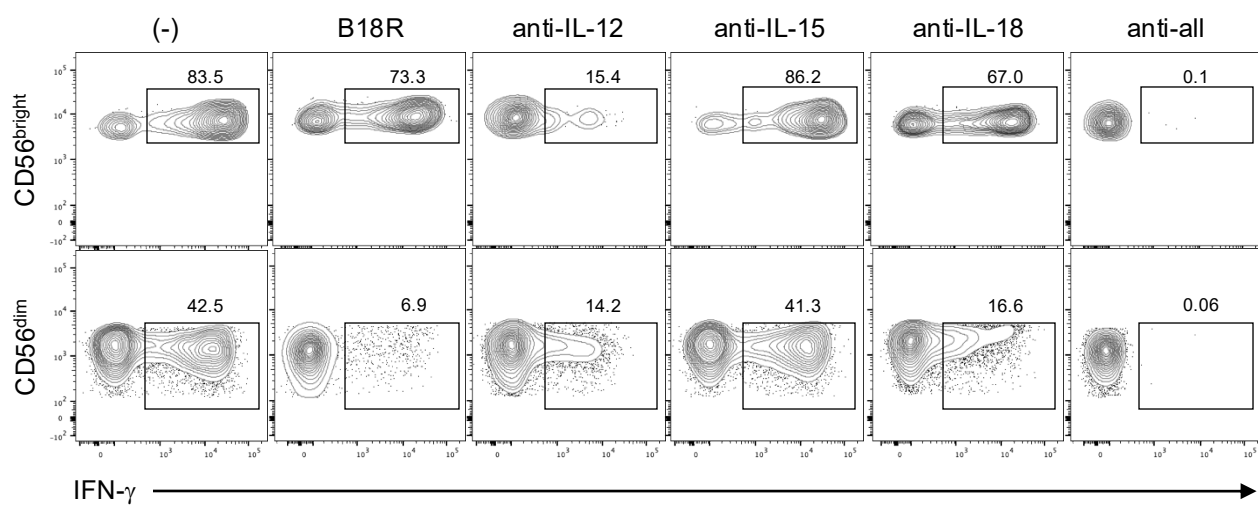

Supplemental Figure 1

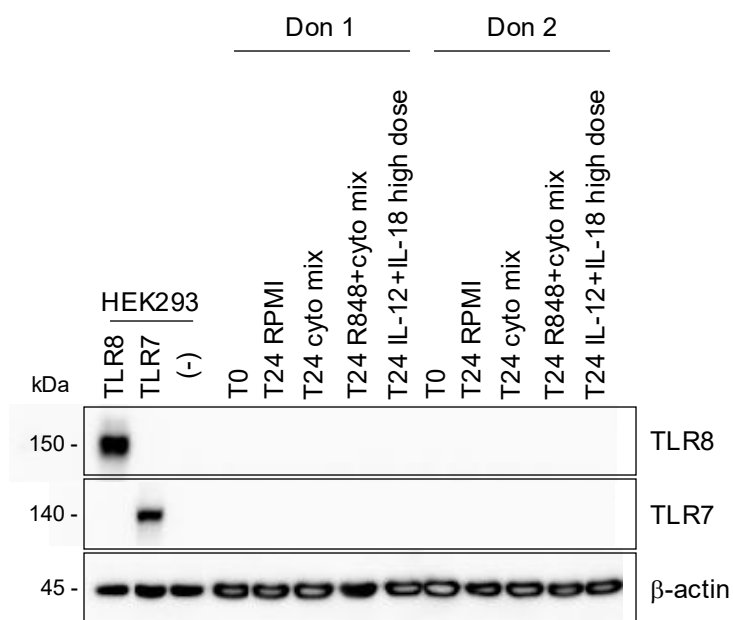

Supplemental Figure 2

Supplement: Supplementary file 1 — Supplementary material 1 [file 12967_2026_7909_MOESM1_ESM.pdf]
