# Supplementary material for: Psoriatic microRNAs induce NK cell activation via an innate immune crosstalk abrogated by the Toll-like receptor 7/8 antagonist Enpatoran
Source: J Transl Med. 2026 Mar 2;24:478. doi: 10.1186/s12967-026-07909-5 (PMC13059162; doi:10.1186/s12967-026-07909-5)
Supplement: Supplementary file 2 — Supplementary material 2 [file 12967_2026_7909_MOESM2_ESM.docx]

**Supplemental Figure 1. IFN-γ production relies on different cytokines in CD56^bright^ and CD56^dim^ NK cell subsets.** Dot plots from one representative experiment showing intracellular staining of IFN-γ in CD56^bright^ (upper panels) and CD56^dim^ (lower panels) NK cells in PBMCs pretreated with specific cytokine blockers (1 μg/ml each) or in combination (anti-all) for 1 hour and then stimulated with pso-miR (-) for 24 hours.

**Supplemental Figure 2. The expression of TLR7/8 by NK cells is not induced upon cytokine nor TLR7/8 stimulation.** NK cells sorted from buffy coats of two healthy blood donors were stimulated as indicated, lysed and subjected to western blot assessment of TLR7, TLR8 and β-actin expression. Stimulation: cytokine mix (2 ng/ml IL-12, 2 ng/ml IL-18, 50 U/ml IFN-α), R848 (1 μg/ml), high dose (5 ng/ml IL-12, 25 ng/ml IL-18). HEK293 transfected with TLR7 and TLR8 were used as positive controls.
